# Peptides-Coated Oncolytic Vaccines for Cancer Personalized Medicine

**DOI:** 10.3389/fimmu.2022.826164

**Published:** 2022-04-14

**Authors:** Sara Feola, Salvatore Russo, Beatriz Martins, Alessandra Lopes, Gaëlle Vandermeulen, Vinciane Fluhler, Camilla De Giorgi, Manlio Fusciello, Sari Pesonen, Erkko Ylösmäki, Gabriella Antignani, Jacopo Chiaro, Firas Hamdan, Michaela Feodoroff, Mikaela Grönholm, Vincenzo Cerullo

**Affiliations:** ^1^ Drug Research Program (DRP) ImmunoViroTherapy Lab (IVT), Division of Pharmaceutical Biosciences, Faculty of Pharmacy, University of Helsinki, Helsinki, Finland; ^2^ Helsinki Institute of Life Science (HiLIFE), University of Helsinki, Helsinki, Finland; ^3^ Translational Immunology Program (TRIMM), Faculty of Medicine Helsinki University, University of Helsinki, Helsinki, Finland; ^4^ Digital Precision Cancer Medicine Flagship (iCAN), University of Helsinki, Helsinki, Finland; ^5^ Advanced Drug Delivery and Biomaterials, Louvain Drug Research Institute, Université Catholique de Louvain, Brussels, Belgium; ^6^ Valo Therapeutics, Helsinki, Finland; ^7^ Department of Molecular Medicine and Medical Biotechnology, Naples University “Federico II”, Naples, Italy

**Keywords:** oncolytic viruses, cancer vaccines, personalized medicine, PeptiCRAd, tumor antigens

## Abstract

Oncolytic Viruses (OVs) work through two main mechanisms of action: the direct lysis of the virus-infected cancer cells and the release of tumor antigens as a result of the viral burst. In this sc.enario, the OVs act as *in situ* cancer vaccines, since the immunogenicity of the virus is combined with tumor antigens, that direct the specificity of the anti-tumor adaptive immune response. However, this mechanism in some cases fails in eliciting a strong specific T cell response. One way to overcome this problem and enhance the priming efficiency is the production of genetically modified oncolytic viruses encoding one or more tumor antigens. To avoid the long and expensive process related to the engineering of the OVs, we have exploited an approach based on coating OVs (adenovirus and vaccinia virus) with tumor antigens. In this work, oncolytic viruses encoding tumor antigens and tumor antigen decorated adenoviral platform (PeptiCRAd) have been used as cancer vaccines and evaluated both for their prophylactic and therapeutic efficacy. We have first tested the oncolytic vaccines by exploiting the OVA model, moving then to TRP2, a more clinically relevant tumor antigen. Finally, both approaches have been investigated in tumor neo-antigens settings. Interestingly, both genetically modified oncolytic adenovirus and PeptiCRAd elicited T cells-specific anti-tumor responses. However, *in vitro* cross-representation experiments, showed an advantage of PeptiCRAd as regards the fast presentation of the model epitope SIINFEKL from OVA in an immunogenic rather than tolerogenic fashion. Here two approaches used as cancer oncolytic vaccines have been explored and characterized for their efficacy. Although the generation of specific anti-tumor T cells was elicited in both approaches, PeptiCRAd retains the advantage of being rapidly adaptable by coating the adenovirus with a different set of tumor antigens, which is crucial in personalized cancer vaccines clinical setting.

## Introduction

Cancer Immunotherapy reprograms a patient´s immune system to generate, stimulate, and sustain specific anti-tumor responses, targeting cancer cells for destruction (1). In the case of a cell-mediated response, the crosstalk between the innate and adaptive arms is essential for generating an optimal anti-tumor T-cell cytotoxic response. In particular, the antigen-presenting cells (APCs), mainly dendritic cells (DCs), are the first players in the frontline of an anti-tumor immune response. Indeed, DCs capture, process, and present tumor antigens (TAs) within the MHC-I complex, priming and/or activating the T-cell response that in turn recognizes and destroys malignant cells (2). All together these elements are key regulators in evoking anti-tumor immune response and so far, a plethora of strategies have been developed to exploit each one of these steps as cancer therapeutic approaches (3). Among the different cancer immunotherapeutic strategies, cancer vaccines based on synthetic peptides have been extensively used to guide the immune response specifically to the eradication of cancer (4, 5). Tumor peptide vaccines have the potential of dramatic anti-tumoral effects due to both strong and anti-tumor-specific immune activation (6); furthermore, immunopeptidomic pipelines to capture the MHC-I peptides landscape is growing and a new avenue to patient´s tailored therapy is on the way (7–9). However, tumor peptides vaccines are still facing major impediments that need to be addressed to reach clinical efficacy (10) and up to date no *in vivo* peptide-based cancer vaccine has obtained FDA approval (10). The main limitations are due to the immunosuppressive tumor microenvironment, self-tolerance, and tumor heterogenicity (10). To overcome these disadvantages and to unleash tumor peptides vaccines’ full potential, several attempts in the field have been made, either combining tumor peptides with adjuvants as Polyinosinic: polycytidylic acid (Poly ICLC) or using genetic-based strategies (i.e., DNA/RNA based vaccines), reaching some level of pre-clinical success but still, a lot of progress needs to be made (10, 11).

Moreover, the breakthrough of immune checkpoint inhibitors (ICIs) targeting programmed death receptor-1 (PD-1), its ligand PD-L1, and cytotoxic T cell-associated antigen 4 (CTL-A4), has increased the full potential of different immune therapeutic cancer treatments, opening new opportunities (12, 13). Indeed, ICIs take the break off of the immune system, unleashing the anti-tumor immune response and/or revitalizing exhausted T-cells (14). The use of ICIs has prolonged the survival of patients affected by highly immunogenic tumors such as metastatic melanoma and lung cancers (15–17); however, the majority of patients still fail to respond to therapy as the effectiveness of ICIs depends on a pre-existing anti-tumor immune response within the TME that can be boosted and/or re-activated by ICIs (18). Thus, combinatorial approaches are needed to induce and/or increase immune components infiltration, turning an immunologically “cold” tumor into a “hot” one (19). Oncolytic virotherapy has been proposed as a platform for the recruitment of immune cells in the TME. Indeed, Oncolytic Viruses (OVs) are a class of viruses genetically modified or naturally occurring able to infect and replicate selectively in cancer cells (20). They are an emerging class of immunotherapeutic agents as in the last decades it became clear that beyond the direct oncolysis, the OVs own a second and more important mechanism of action based on inducing immunogenic cell death (ICD) that ultimately activate the immune system (20, 21). Upon oncolytic cell burst, tumor-associated antigens (TAA) and eventually neoantigens (TNA) are released and ingested by DCs that in turn prime and activate specific anti-tumor T cells. Moreover, the oncolytic cell burst promotes immunogenic cell death (ICD), with the release of several cellular factors known as damage-associated molecular patterns (DAMPs) such as calreticulin (ecto-CRT), secreted adenosine triphosphate (ATP) and high mobility group box 1 protein (HMGB1), enhancing the anti-tumor immunity (22–24). In addition to that, OV mediated cell lysis is combined with the accumulation of viral components such as nucleic acid (DNA, dsRNA, ssRNA, and 5′-triphosphate RNA), proteins and capsid components named pathogen-associated molecular patterns (PAMPS) (20, 22). In turn, DAMPs and PAMPs license the DCs to bolster the generation of an immunogenic response instead of a tolerogenic one (21).Nevertheless, the anti-viral T cells response is predominant in the immune reaction, with only a minor component of this latter being a specific anti-tumor response.

To take full advantage of the natural immunogenicity of OVs for one side and to exploit the specificity of tumor peptide vaccines to guide the tumor response for cancer´s eradication on the other side, genetically modified OVs expressing tumor antigens (TA) has been extensively produced and tested. However, expensive and time-consuming protocols are required to generate OVs encoding one or more TAs and these requirements are not compatible with patient-tailored treatment; additionally, as the viral genome encodes the TAs, their production demands robust infection and therefore the final vaccinal outcome depends on the unpredictable and highly variable intrinsic sensitivity of each tumor to OVs (25). To avoid this main disadvantage and to increase the anti-tumor immune response, PeptiCRAd a technology that consists of an oncolytic adenovirus (OAd) decorated with MHC-I tumor peptides has been developed and is currently being explored as a cancer therapeutic vaccine. The technology uses poly-lysine tail-peptides that through electrostatic interactions bind the adenoviral capsid; a reaction takes only 15 minutes (26).

In this work, we aimed to investigate and compare OVs encoding TAs and PeptiCRAd with main regards to their cancer prophylactic and therapeutic efficacy. Herein, we have generated an oncolytic adenovirus (OAd) encoding either the model protein chicken ovalbumin (OVA) or the more clinically relevant tumor antigen murine tyrosinase-related protein 2 (TRP_2_); then we have carefully characterized the T-cell immune profile in mice pre-immunized either with the OAd encoding the TAs or with PeptiCRAd. Next, we have challenged the therapeutic efficacy of PeptiCRAd and cloned viruses by treating mice bearing the established B16.OVA tumor model. Finally, we have moved to a more complex and clinically relevant setting to mimic the heterogeneous tumor profile. To this end, we have generated OAd encoding previously described neoantigens in the poor immunogenic model B16F1 (27) to compare the anti-tumor efficacy of PeptiCRAd coated with the same set of neoantigen.

In this study, we have shown that PeptiCRAd technology is as efficient as OAd engineered to express TAs; however, translated in a clinical scenario, PeptiCRAd retains the advantage of being easily adaptable for personalized cancer therapy, bypassing the need of engineering cancer-specific patient OVs.

## Material and Methods

### Cell Lines and Reagents

Human lung carcinoma cell line A549, human triple-negative breast cancer cell line MDA-MB-436, and human ovarian cancer SKOV-3 cell line were cultured in DMEM supplemented with 10% FBS (Gibco), 1% glutamine (GIBCO), 100 μg/ml streptomycin, and 100 U/ml penicillin (Life Technologies, California). Human epithelial colorectal adenocarcinoma CACO-2 cell line was cultured in DMEM supplemented with 20% FBS, 1% glutamine, 100 μg/ml streptomycin, and 100 U/ml penicillin. Murine colon cancer cell line CT26 was cultured in RPMI supplemented with 10% FBS, 1% glutamine, 100 μg/ml streptomycin, and 100 U/ml penicillin. Murine triple-negative breast cancer cell line 4T1 was cultured in RPMI high glucose. Human melanoma cell line SK-MEL2 was cultured in EMEM supplemented with 10% FBS, 1% glutamine, 100 μg/ml streptomycin, and 100 U/ml penicillin. Murine dendritic cell line JAWSII was cultured in alpha MEM supplemented with 20% FBS, 1% glutamine, 100 μg/ml streptomycin, and 100 U/ml penicillin. All the cell lines were purchased from ATCC. B16F1, a melanoma cell line from C57BL/6 mice, was kindly provided by Professor Veronique Preat (Université Catholique de Louvain, Belgium). B16F1 was cultured in MEM complete medium, containing 10%FBS, 100 μg/ml streptomycin, and 100 U/ml penicillin.

B16.OVA, a mouse melanoma cell line expressing chicken OVA, was kindly provided by Professor Richard Vile (Mayo Clinic, Rochester, MN, USA). B16.OVA cells were cultured in RPMI with 10% FBS, 1% L-glutamine, 1% penicillin/streptomycin, and 5 mg/mL Geneticin (Life Technologies). The cells were cultivated at 37°C, 5% CO_2_ in a humidified atmosphere. The following peptides were used through the study and were purchased from Zhejiang Ontores Biotechnologies (Zhejiang, China):

DSGSPFPAAVILRDALHMARGLKYLHQ(PbK),

PSKPSFQEFVDWENVSPELNSTDQPFL (Kif18b), EFKHIKAFDRTFADNPGPMVRPWQSAS(Cpsf3l),

WNRQLYPEWTEAQRL (gp100) SVYDFFVWL (TRP2), KKKKKDSGSPFPAAVILRDALHMARGLKYLHQ (PbK),

KKKKKKKKKKPSKPSFQEFVDWENVSPELNSTDQPFL (Kif18b)

KKKKKEFKHIKAFDRTFADNPGPMVRPWQSAS (Cpsf3l)

KKKKKKWNRQLYPEWTEAQRL (gp100)

KKKKKKSVYDFFVWL (TRP2)

SIINFEKL (OVA)

KKKKKKSIINFEKL (OVA)

### IFN-γ ELISpot

IFN-γ ELISpot assays were performed using a commercially available mouse ELISpot reagent set (ImmunoSpot, Bonn Germany) and 20 ng/uL of each peptide was tested in *in vitro* stimulations of 3x10^5^ splenocytes for each well at 37 °C for 72h. Spots were counted using an ELISpot reader system (ImmunoSpot, Bonn Germany).

### INF-γ/IL-10 FluoroSpot

INF-γ/IL-10 FluoroSpot was performed using a commercially available mouse FluoroSpot reagent set (Mabtech, Nacka Strand, Sweden) and 20 ng/uL of each peptide was tested in *in vitro* stimulations of 3x10^5^ splenocytes for each well at 37 °C for 72h. Spots were counted using a FluoroSpot reader system (Nacka Strand, Sweden).

### Quantification Assay for Ad-OVA

Human lung carcinoma A549 cells, human TNBC MDA-MB-436 cells, and human epithelial colorectal adenocarcinoma CACO-2 were infected with 10MOI, and the supernatant was collected at 24h post-infection. Murine TNBC 4T1 cells and murine CT26 colon cell line were infected with 500 MOI and the supernatant was collected at 48h and72h post-infection. The Human lung carcinoma A549 cell line was infected with 10 MOI and the cell pellet was collected at 48h post-infection. The cell lysate and the supernatants were analyzed for the presence of OVA by ELISA (ABIN2537475, Antibodies) according to the manufacturer´s instructions.

### PeptiCRAd Complex Formation

The PeptiCRAd complex was prepared by mixing the oncolytic adenovirus and each peptide with a polyK tail. We mixed polyK-extended epitopes with Ad5/3D24 for 15 minutes at room temperature before treatments with the PeptiCRAd complexes. More details about the stability and formation of the complex can be found in our previous study (26).

### Cross-Presentation Experiment

Murine dendritic JAWS-II cells were infected with 250 MOI of different viruses (Ad5/3-D24, Ad5/3-CMV-OVA, and PeptiCRA-SIINFEKL). One well was left uninfected as control. After 4 hours of incubation, the medium was changed and at 24h and 48h post-infection the cells were stained.

### Animal Experiment

All animal experiments were reviewed and approved by the Experimental Animal Committee of the University of Helsinki and the Provincial Government of Southern Finland (license number ESAVI/11895/2019). 4-6 weeks old female C57BL/6JOlaHsd mice were obtained from Envigo (Laboratory, Bar Harbor, Maine UK).

For the prophylactic experiment, mice (n=10 per group) were allocated in different groups according to the treatment and each mouse was subcutaneously injected with 1x10^9^ VP (viral particle). The prime and boosting were done respectively on days 1,2,3 and 10 and the mice were sacrificed on day 14. For the B16.OVA tumor-bearing mice experiment, 3x10^5^ B16.OVA cells were injected subcutaneously. Details about the schedule of the treatment can be found in the figure legends. For the B16F1 tumor-bearing mice experiment, 1x10^5^ and 0.5 x10^5^cells were injected subcutaneously on the right and left flank of the mice respectively.

The viral dose was 1x10^9^ VP/tumor complexed with 20 µg of a single peptide or with 4 µg+4 µg 4 µg+4 µg +4 µg mixture of five peptides.

### Oncolytic Adenovirus

In this study, the virus Ad-OVA, Ad-TRP2, and Ad-Epitopes were used and they were generated according to Hamdan et al. (28). Briefly, Ad-OVA, Ad-TRP2, and Ad-Epitopes are conditionally replicating adenovirus serotype 5 with adenovirus 3 fiber knob modification and 24-base pair deletion of the gene E1A. The CR1-alpha and gp19Kgenes of the E3A region were replaced with human cytomegalovirus (CMV) promoter region and OVA (Ad-OVA), murine TRP2 (Ad-TRP2), or five epitopes (Ad-Epitopes). The five epitopes are expressed as single peptides separated through a linker of arginine. The cloning cassette have been inserted in E3 adenoviral region. A rabbit β-globin polyadenylation signal was added. The VP concentration was measured at 260nm, and infections units (IU) were determined by immunocytochemistry (ICC) by staining the hexon protein in infected A549 cells.

### Cell Viability Assays

Human SK-MEL-2 cells, human lung carcinoma A549 cells, human TNBC MDA-MB-436 cells, murine TNBC 4T1 cells, and murine melanoma B16.OVA and B16F1 cells were infected with various amounts of Ad-OVA, Ad-TRP2, Ad-Epitopes, and Ad5/3-D24 or left uninfected. Cells were analyzed for their viability3 and 5 days postinfection with the CellTiter 96 AqueousOne Solution MTS Reagent (Promega), according to the manufacturer’s instructions, and a multi-well plate reader (Varioskan Flash; Thermo Labsystems) was used to determine the absorbance of the samples.

### Flow Cytometry

The following antibodies were used in the cross-presentation experiments: TruStain Fcblock anti-mouse CD16/32 (101320; BioLegend), FITC-CD11c (117306; Biolegend), APC-H2Kb-bound SIINFEKL (141606; Biolegend), APC/Cy7-CD40 (124637; Biolegend), PerCP/Cy5.5- ICAM_1 (116123; Biolegend), BV510-CD86 (563077; BD), PE/Cy7-MHC-II (107629; Biolegend), V450-CD80 (12519; BD). The following antibodies were used for the immunological analysis in the *in vivo* animal experiments: FITC-CD8 (553062; BD), PE-CD4 (100408; Biolegend), APC-CXCR3 (562266; BD), PerCP/Cy5.5-CD3 (100732; Biolegend), PE-CXCR3 (155903); FITC-CD8 (11083782); PerCP/Cy5.5-CD3 (100328);. Flow cytometric analyses were performed using Fortessa LSR Flow Cytometer (BD Biosciences) or BD Accuri C6 Plus (BD Bioscience). FlowJo software v.10 (FlowJo) was used for the data analysis.

### qPCR Analysis

B16F1 cell lines were collected 48 hours after infection and the RNA was extracted by using the Rneasy Plus Mini kit (74134, Qiagen), according to the protocol provided by the manufacturer. The RNA extraction quality was checked by electrophoresis in 1% agarose gel. The RNA samples were reverse-transcribed by SuperScript-IV Reverse Transcriptase (18091050, Invitrogen) and random hexamers as primers, according to the protocol provided by the manufacturer. The reverse transcription products were used as DNA templates for PCR reactions. The Real-Time PCR was performed using StepOnePlus _Real Time PCR system (Applied Biosystems). The qPCR experiment was designed as C_T_ comparative experiment (ΔC_T_). As the fluorescent dye, SYBR Green was used (SYBR_Green PCR master mix, A25742, Applied Biosystem). Two reaction master mixes were prepared for two pairs of primers to amplify murine GAPDH and the epitopes, following the protocol provided by the manufacturer. 10 ng of cDNA as template and 200 nM of the primers were used for each reaction.

### RT-PCR

Human lung adenocarcinoma A549 cells and murine TNBC 4T1 cells were infected with 5 and 100 MOI of Ad5/3-D24, Ad5/3-D24-CMV-TRP2, and Ad5/3-D24-CMV-OVA. pCMV-OVA was transfected as well as control with Lipofectamine (Lipofectamine 2000) according to the manufacturer’s instructions The cells were collected 48 hours post-infection and the RNA was extracted by using the RNeasy Plus Mini kit (74134, Qiagen), according to the protocol provided by the manufacturer. The RNA samples were reverse-transcribed by SuperScript-IV Reverse Transcriptase (18091050, Invitrogen) and random hexamers as primers, according to the protocol provided by the manufacturer. The reverse transcription products were used as DNA templates for PCR reactions.

### Statistical Analysis

Statistical analysis was performed using GraphPad Prism 9.0 software (GraphPad Software Inc.). Details about the statistical tests for each experiment can be found in the corresponding figure legends.

## Results

### *In Vitro* Characterization of Oncolytic Adenovirus Encoding Model Tumor Antigens

**Figure 1:**
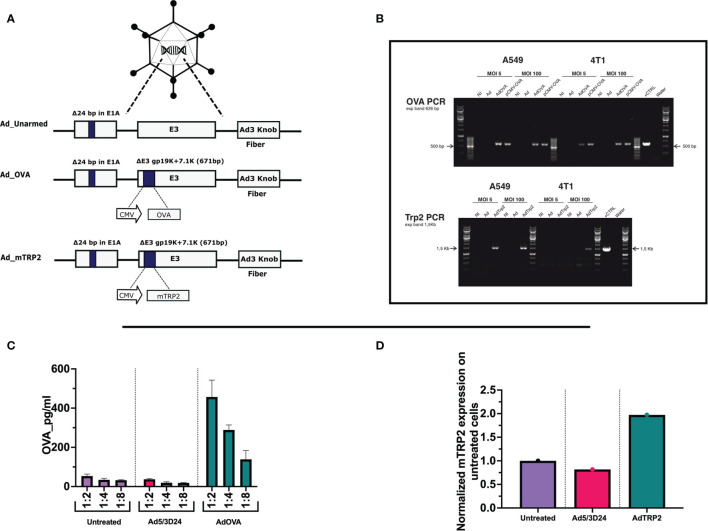
Generation and characterization of AdOVA and AdTRP2 **(A)** Schematic representation of oncolytic adenovirus delta 24 (Ad5/3-D24) constructs with modifications in E1, E3, and fiber region. Both unarmed (Ad_unarmed) and armed (Ad_OVA and Ad_mTRP2) bear a deletion of 24bp in the E1A gene. Additionally, AdOVA (Ad_OVA) and AdTRP2 (Ad_mTRP2) contains an expression cassette under CMV promoter in E3 region. **(B)** Reverse-transcribed PCR reaction with specific primers for OVA and mTRP2 on cDNA derived from A549 and 4T1 infected with 5 and 100 MOI. **(C, D)** A549 cells were infected with 10MOI and the cell lysate was collected at 48h and OVA and TRP2 levels were checked by ELISA and the data are depicted as bar plots.

The use of OVs encoding TAs has been extensively explored as cancer vaccines to take full advantage of both viral immunogenicity and tumor peptides that direct the specificity of the anti-tumor adaptive immune response. However, the vaccinal outcome heavily depends on the viral infectivity and consequently viral replication as each tumor owns a different sensitivity to OVs. To bypass this limitation, we wanted to investigate whether the PeptiCRAd technology, based on OAd decorated with tumor antigens, could be considered a legitimate alternative cancer vaccine to OAd encoding TAs. To this end, we needed to generate OAds encoding TAs to be used for comparative studies. Therefore, we cloned OAds encoding the tumor model chicken ovalbumin (OVA) and the more relevant tumor antigen murine tyrosinase-related protein 2 (TRP_2_) by applying GAMER-Ad protocol according to Hamdan et al. (28). Briefly, the region E3 was removed and replaced with the gene of interest (GOI) contained in E3 deleted of gp19K and 7.1K (**Figure 1A**). After constructing the adenovectors expressing the GOI, we digested and transfected the construct in A549, and we waited for the viral plaque formation. Once the plaques appeared, the viruses were harvested and purified. Next, we proceeded with an extensive validation of the generated novel OAd encoding TAs. First, we assessed whether the oncolytic fitness or replication of the viruses was affected upon the insertion of the GOI in the viral genome. The viruses have a 24-bp deletion in the E1A region conditioning such

viruses to replicate only in Retinoblastoma (Rb)-deficient cells as the majority of human cancer cell lines are. Moreover, the OAd used through the study is serotype 5 with adenovirus 3 fiber knob modification, allowing the infection of a wider range of cells unrestrictedly to CAR receptor expression (29). Hence, lung carcinoma cells (A549), ovarian cancer cells (SKOV3), triple-negative breast cancer cells (MDA-MB-436), melanoma cells (SKMEL-2) were infected with unarmed Ad5/3Δ24, AdOVA, and AdTRP2 viruses. Oncolysis was observed at days 3 (**Supplementary Figures 1A–D**) and 5 (**Supplementary Figures 2A–D**) post-infection in a dose-dependent fashion. In all the analyzed cell lines, the oncolysis levels of the cloned viruses resembled those of the unmodified virus, indicating that the transgene did not affect oncolytic potency or virus replication. To further corroborate this, two murine cell lines, B16.OVA and 4T1 were also investigated. Human adenoviruses serotype can infect murine cell lines but are unable to replicate. As expected, no cell death was observed with either murine cell line when infected with the unmodified, AdOVA and AdTRP2 viruses, at day 3 (**Supplementary Figures 1E, F**) and day 5 (**Supplementary Figures 2E, F**) post-infection, showing that oncolytic fitness or replication was unaltered in the cloned viruses. We then investigated the transgene expression at both RNA and protein levels. Reverse transcription PCR confirmed the presence of mRNA expression for OVA in one human cell line (A549) and one murine cell line (4T1) infected with 5 and 100 MOI of AdOVA (**Figure 1B**). The presence of TRP_2_ was confirmed in A549 at both 5 and 100MOI, whereas in 4T1, we detected the presence of 4T1 at 100MOI (**Figure 1B**). Moreover, the cell lysate from A549 infected with the cloned viruses was investigated for the presence of OVA and TRP_2_. The analysis showed the expression at the protein level for both OVA (**Figure 1C**) and TRP_2_ (**Figure 1D**). Moreover, the supernatant of human A549, MDA-MB-436, and CACO2 cell lines infected with AdOVA confirmed the protein expression of OVA (**Supplementary Figures 3A-C**); the results were confirmed in murine 4T1 and CT26 cell line AdOVA infected at 48h and 72h post-infection (**Supplementary Figures 3D, E**). Overall, the data confirmed that the insertion of the GOI in the viral genome permitted normal oncolytic activity and that the viruses expressed the transgene as demonstrated at both mRNA and protein levels, granting their use for further comparative analysis.

### Peptides-Coated Platform Showed Immunogenic Activity Comparable to OAd Encoding TAs in *In Vitro* and *In Vivo* Studies

**Figure 2:**
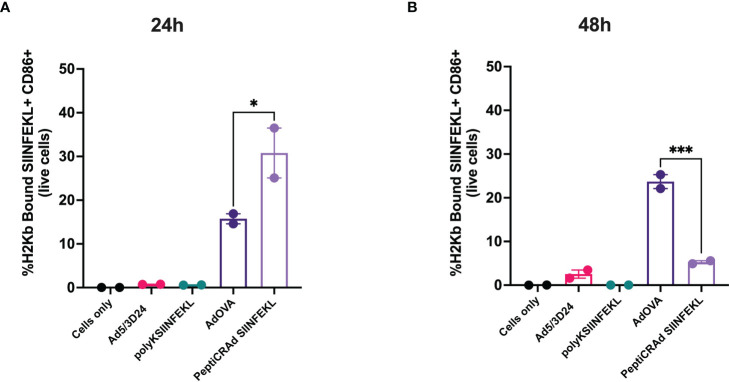
DCs cross-present antigen in an immunogenic fashion upon stimulation with Ad encoding TAs or PeptiCRAd. Mouse dendritic cell line JAWS II was pulsed with Ad5/3Δ24, peptide alone (polyKSIINFEKL), AdOVA, PeptiCRAd-SIINFEKL or left unpulsed (cells only). The viruses were used at 250 MOI. Flow cytometry analysis was used to determine the cross-presentation at 24h **(A)** and 48h **(B)** post incubation. CD86 was used to measure DCs activation and an antibody specific for OVA peptide SIINFEKL complexed with H2Kb to detect the antigen presentation. The data are depicted as bar plot mean ± SEM. Statistical analysis was performed with ordinary One-way ANOVA (ns P > 0.05, *P ≤ 0.05, **P ≤ 0.01, ***P ≤ 0.001, P ≤ 0.0001).

**Figure 3:**
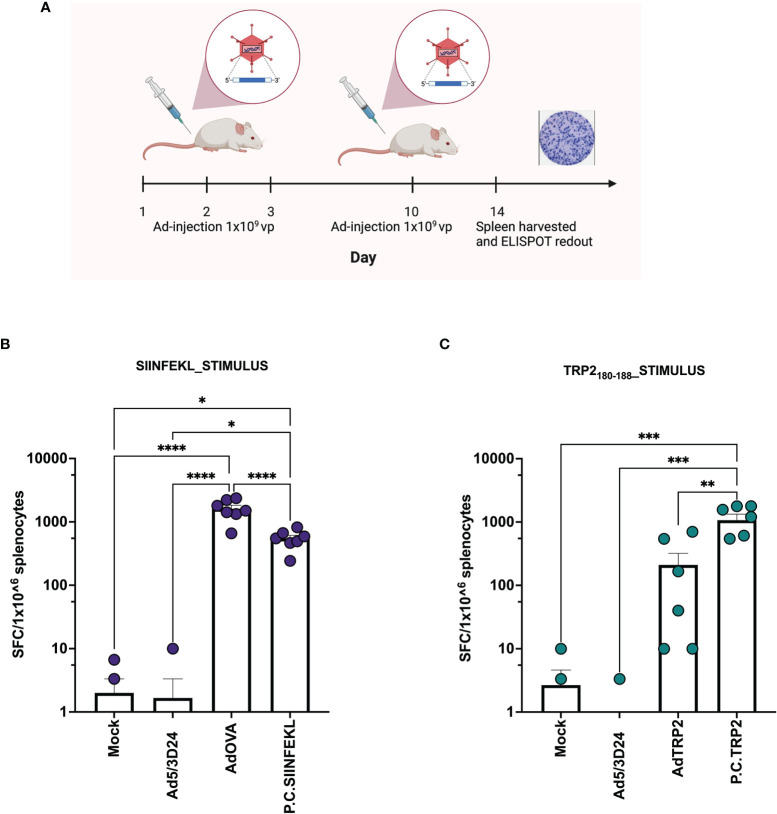
Ad encoding TAs and PeptiCRAd show *in vivo* prophylactic efficacy. **(A)** Schematic representation of the schedule followed during the vaccination procedure. The mice have been subcutaneously injected with Ad5/3Δ24, PeptiCRAd (P.C. SIINFEKL, P.C. TRP2), cloned viruses (AdOVA, AdTRP2) or PBS (Mock) at day 1,2,3 and 10. The spleens were harvested at day 14. **(B, C)** IFN-γ ELISpot was performed on harvested splenocytes and individual response to SIINFEKL **(B)** and TRP2 **(C)** for each mouse is reported as IFN**-**γ spot forming cells (SFC)/10^6^ splenocytes. The data are depicted as bar plot and mean + SEM is shown. (P.C.=PeptiCRAd) and the statistical analysis was performed with ordinary One-way ANOVA (ns P > 0.05, *P ≤ 0.05, **P ≤ 0.01, ***P ≤ 0.001, ****P ≤ 0.0001). Created with BioRender BioRender.com.

After the generation and characterization of AdOVA and AdTRP2, we used them as a benchmark as we carefully sought to characterize the immunological effects of PeptiCRAd. First, a comparative immunogenic analysis of PeptiCRAd and the cloned viruses was performed as regards APCs activation and antigen presentation. To this end, we used the murine dendritic cell line JAWS-II. As Ovalbumin is broadly studied and several tools are available for research purposes, the first comparative analysis was done exploiting AdOVA and the PeptiCRAd counterpart. Hence, JAWS-II cells were pulsed either with AdOVA or PeptiCRAd coated with the OVA epitope SIINFEKL; cells incubated with peptide or virus alone were used as controls. Then we stained the cells at 24h and 48h post-incubation with a monoclonal antibody specific for the OVA peptide SIINFEKL complexed with H2Kb. An example of the gating strategy is shown in **Supplementary Figure 4**. To determine whether the DCs were presenting the antigen in a tolerogenic or stimulatory manner, activation markers (CD80, CD86, MHC-II, CD40) were included in the analysis as well. Interestingly, at 24h post-incubation, H2Kb bound to SIINFEKL and the co-stimulatory factor CD86 were upregulated in JAWS-II treated either with PeptiCRAd-SIINFEKL or with AdOVA (**Figure 2A**). Instead, CD40 (**Supplementary Figure 5A**), CD80 (**Supplementary Figure 5B**), MHC-II (**Supplementary Figure 5C**) and the adhesion molecule ICAM-I (**Supplementary Figure 5D**) showed comparable expression among the different treatment groups. Additionally, we observed that in PeptiCRAd-SIINFEKL treated DCs the level of H2Kb bound to SIINFEKL and CD86 were higher compared to JAWS-II treated with AdOVA. Indeed, in DCs treated with PeptiCRAD-SIINFEKL, the expression of the H2Kb bound to SIINFEKL and CD86 markers were already high at 24h post-incubation, whereas AdOVA induced comparable levels of the H2Kb bound to SIINFEKL and CD86 at 48h post-incubation (**Figure 2B**). These results match the different kinetics of peptides versus proteins, with the first being directly available to DCs presentation and the latter depending on the vector expression and protein processing. Moreover, at 48h post-incubation CD40 (**Supplementary Figure 5E**), CD80 (**Supplementary Figure 5F**), MHC-II (**Supplementary Figure 5G**), and the adhesion molecule ICAM-I (**Supplementary Figure 5H**) showed similar expression pattern among the different treatment groups, whereas CD80 (**Supplementary Figure 5F**) was upregulated in the adenovirus treated cells. Next, as we wanted to characterize the *in vivo* efficacy of PeptiCRAd as a prophylactic vaccine in comparison to Ad encoding TAs, mice were immunized by subcutaneous direct injection of Ad-OVA and AdTRP2 and with counterpart coated peptide technology according to the schematic depicted in **Figure 3A**. Spleens were harvested at the end of the pre-immunization procedure and the T cell-specific immune response was functionally characterized by IFN-γ ELISPOT assay upon stimuli such as SIINFEKL and TRP2. Our data showed that both cloned viruses and PeptiCRAd induced T cell-specific response (**Figures 3B, C**) *in vivo*, confirming the stimulatory activity observed in *in vitro* experimental settings. Overall, the first set of results showed that peptide-coated oncolytic vaccine PeptiCRAd activates APCs that in turn prime and induce specific T cell response well in line with the established outcome of OAd encoding TAs used as cancer therapeutic vaccine.

### Peptide-Coated Cancer Vaccine and OAd Encoding TAs Showed Similar Therapeutic Activity in a Syngeneic Mouse Model of B16.OVA Melanoma

**Figure 4:**
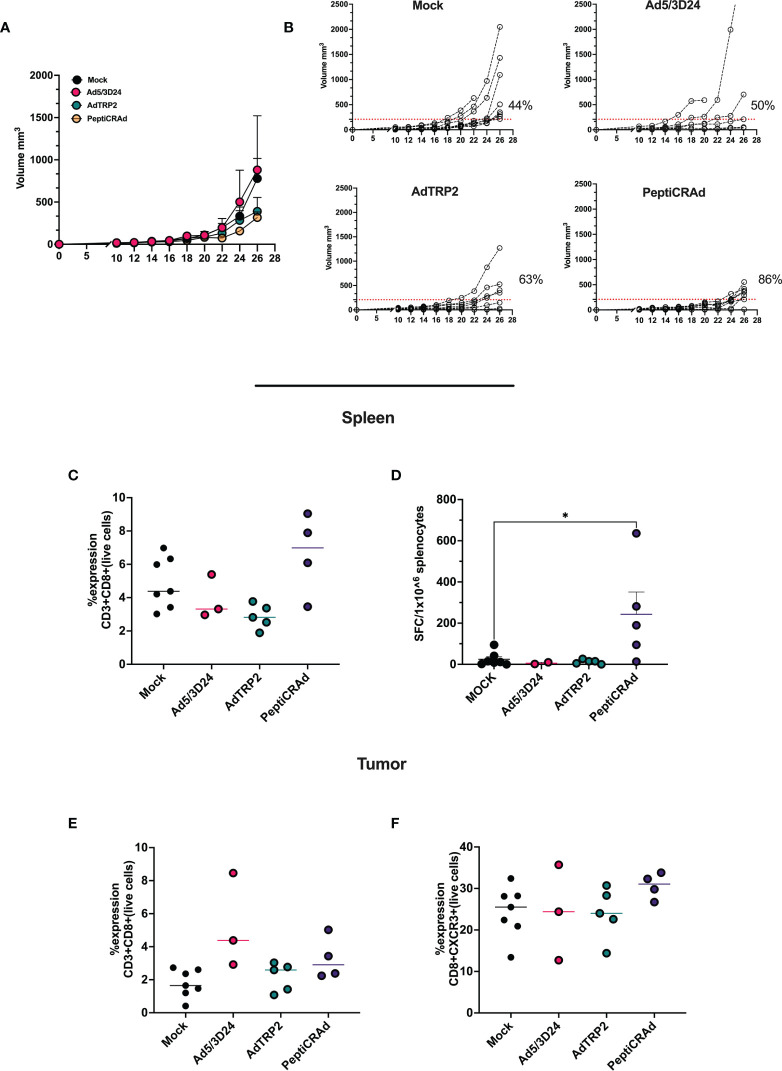
Intratumorally administration of PeptiCRAd induced tumor regression and immunological modulation **(A)** Ad5/3Δ24, AdTRP2 or PeptiCRAd was given intratumorally at 9,11,13 and 15 days post tumor implantation. The tumor growth was followed until the end of the experiment and the tumor size is presented as the mean ± SEM. Statistically difference was assessed with two-way ANOVA (ns P > 0.05, *P ≤ 0.05, **P ≤ 0.01, ***P ≤ 0.001, P ≤ 0.0001). (Mock n=9, Ad5/3Δ24 n=6 AdTRP2 n=8 or PeptiCRAd n=7) **(B)** Single tumor growth for single mouse for each treatment group is depicted. A threshold of 209 mm^3^ was set to define the percentage of mice responding to the different therapies (red dotted line). The percentage of responders in each treatment group is shown on the right side of the dotted line. (The threshold was defined as the median of the tumor size at the last day of the experiment in the Ad5/3Δ24 treated group). **(C)** Flow cytometry analysis of spleens from treated mice showing the frequency of CD8+T cells. Data are expressed as single dot for each mouse and median is reported **(D)** IFN-γ ELISpot was performed on harvested splenocytes and individual response to TRP2 for each mouse is reported as IFN**-**γ spot forming cells (SFC)/10^6^ splenocytes. The data are depicted as dot plot and mean ± SEM is shown. Statistical analysis was performed with ordinary One-way ANOVA (ns P > 0.05, *P ≤ 0.05, **P ≤ 0.01, ***P ≤ 0.001, P ≤ 0.0001). The frequency of CD8+ **(E)** and CD8+CXCR3+ **(F)** was analyzed in TME through flow cytometry analysis and the data are shown as single dot for each mouse and median is reported.

As aforementioned, PeptiCRAd and OAd encoding TAs exerted similar immunological activity in both *in vivo* and *in vitro* experimental settings. These data prompted us to assess the therapeutic efficacy of PeptiCRAd compared to OAd encoding TAs in a poorly immunogenic tumor model. To this end, immunocompetent C57Bl/6 mice were subcutaneously injected with the syngeneic B16.OVA melanoma tumor cells in the right flank. When the tumors were established, the mice were intratumorally treated either with AdTRP2 or the PeptiCRAd counterpart. Mice injected only with PBS (Mock) and Ad5/3Δ24 groups were used as controls; the viral dose used was 1x10^9^ VP/tumor. PeptiCRAd and AdTRP2 improved tumor growth control (**Figure 4A**) as depicted also in the single tumor growth per mouse per each treatment group, with 63% and 86% of responders respectively in AdTRP2 and PeptiCRAd treated mice (**Figure 4B**). Next, we sought to investigate the immunological modulation due to different therapeutic regimens. First, we observed an increase in the CD8+ T cell population in spleens of mice treated with PeptiCRAd (**Figure 4C**) compared to other groups; to dissect the functional profile of those CD8+ T cells, an INF-γ ELISPOT assay was performed on the spleen of the treated mice; upon TRP2 stimulus the production of IFN-γ increased significantly in PeptiCRAd treated mice, highlighting systemic generation of specific anti-tumor T cells following PeptiCRAd treatment (**Figure 4D**). Moreover, CD8+ T cells showed an increasing trend within the tumor microenvironment (**Figure 4E**) in all the groups that underwent adenovirus-based treatment (Ad5/3Δ24, AdTRP2, and PeptiCRAd); however, a tendency in increased effector phenotype CXCR3+ among the CD8+ T cells was reported only in PeptiCRAd treated group (**Figure 4F**).

Overall, the data confirmed that PeptiCRAd technology could be a valid alternative to OAd encoding TAs; from an immunological point of view, PeptiCRAd elicited specific anti-tumor T cells response in secondary lymphoid organs, additionally inducing an increased tumor infiltration of effectors phenotype CD8+T cells.

### PeptiCRAd as Prophylactic Vaccine-Induced Specific Anti-Tumor Immune Response Addressing Tumor Heterogeneity

**Figure 5:**
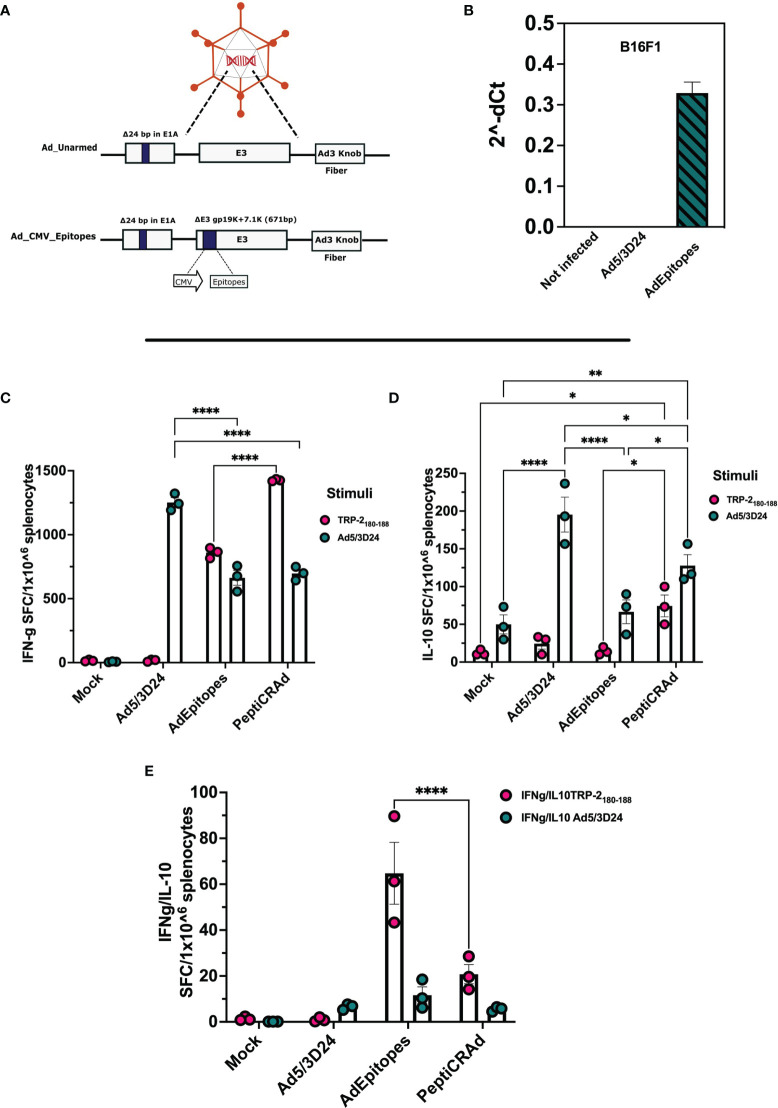
Generation and characterization of AdEpitopes **(A)** Schematic representation of oncolytic adenovirus delta 24 (Ad5/3-D24) constructs with modifications in E1, E3, and fiber region. Both unarmed (Ad_unarmed) and armed (Ad_CMV_Epitopes) bear a deletion of 24bp in the E1A gene. Additionally, AdEpitopes (Ad_CMV_Epitopes) contains an expression cassette under CMV promoter in E3 region. **(B)** Real-time PCR was performed in B16F1 infected with 5MOI of AdEpitopes, Ad5/3Δ24 or left untreated (not infected) and the fold gene expression is analyzed as 2^-dCt^.The data are represented ad bar blot and mean ± SEM. **(C)** IFN-γ ELISpot was performed on harvested splenocytes from mice treated with Ad5/3Δ24, AdEpitopes or PBS (Mock). The individual response to TRP2 (pink) and Ad5/3Δ24 (green) is reported as IFN**-**γ spot forming cells (SFC)/10^6^ splenocytes. **(D)** IL-10 FluoroSpot evaluated the level of IL-10 released upon stimulation with TRP2 (pink) and Ad5/3Δ24 (green) in splenocytes harvested from mice immunized with Ad5/3Δ24, AdEpitopes or PBS (Mock). **(E)** The ratio IFN**-**γ/IL-10 spot forming cells is depicted and TRP2 (pink) and Ad5/3Δ24 (green). The data are shown as bar and dot plot for each technical replicate, and mean ± SEM. Significance was assessed with ordinary One-way ANOVA (ns P > 0.05, *P ≤ 0.05, **P ≤ 0.01, ***P ≤ 0.001, ****P ≤ 0.0001).

After demonstrating that PeptiCRAd technology could be used as a treatment alternative to OAd encoding TAs as regard both prophylactic and therapeutic efficacy by exploiting the tumor model antigens OVA and TRP2, we aimed to challenge our technology to address the complex tumor heterogeneity. Indeed, upon mutations, the immunopetidomic landscape of cancer cells changes and novel mutated epitopes named tumor neo-antigens (TNAs) are presented within the MHC-I complex (30). These latter are preferential targets for cancer treatment as they bypass the negative T-cell clonal selection (31). Moreover, to engage both arms of adaptive immune response (CD8 and CD4), we included in the subsequent experimental setting also MHC-II restricted epitopes. Hence, we sought to investigate whether PeptiCRAd could exert immunological modulation also in the context of MHC-I and MHC-II TNAs, benchmarking our technology by using OAd encoding TNAs. To this end, we have first generated an OAd encoding five different neo-epitopes (AdEpitopes) previously described in the B16F1 melanoma model (PbK, Kif18b, Cpsf3l, gp100, and TRP_2_) (27). Briefly, to generate the AdEpitopes we used the GAMER-Ad protocol described in Hamdan et al. (28), removing the region E3 and replacing it with the GOI a poly-epitopes construct contained in E3 deleted of gp19K and 7.1K (**Figure 5A**). To assess whether the insertion of the transgene could interfere with the oncolytic activity of the cloned virus, we infected the human lung (A549) cancer cell line, the human triple-negative breast (MDA-MB-436) cancer cell line, the human ovarian (SKOV3) cancer cell line and the human melanoma (SKMEL-2) cancer cell line with different amount of MOI and we checked the cell viability at day 3 (**Supplementary Figures 6A-D**) and 5 (**Supplementary Figures 7A–D**) post-infection. The cloned virus showed oncolytic activity accordingly to MOI concentration, resembling the unarmed OAd (Ad5/3D24) and thus confirming that the presence of the GOI in the viral genome was compatible with the adenoviral replication and oncolytic activity. Additionally, two murine cell lines (4T1 and CT26) were infected at different MOI and the cell viability was checked as well at day 3 (**Supplementary Figures 6E, F**) and 5 (**Supplementary Figures 7E, F**) post-infection. The cell viability was stable during the infection, confirming that human OAd oncolytic activity is restricted to the human setting. Next, we analyzed the expression of GOI in the B16F1 cell line infected with 5 MOI of the cloned virus. Cells not infected and cells infected with unarmed virus (Ad5/3Δ24) were used as control. RNA was extracted from B16F1, and real-time quantitative reverse transcriptase-polymerase chain reaction (qRT-PCR) was performed; the results confirmed the presence of the transgene in the transfected cells at RNA level (**Figure 5B**). Next, we wanted to assess the prophylactic activity of PeptiCRAd decorated with the neoepitopes in comparison to the cloned virus. Hence, mice were pre-immunized either with the cloned virus or with PeptiCRAd counterpart, and the spleens were harvested at the end of the pre-immunization protocol to investigate the T cell response induced following the different regiments administrated. The IFN-γ ELISPOT assay showed specific TRP2 T-cell response in both PeptiCRAd and AdEpitopes preimmunized mice (**Figure 5C**) and interestingly PeptiCRAd inducted a statistically higher amount of specific T cells compared to AdEpitopes (**Figure 5C**); even though the adenoviral T-cells response was observed in all the groups adeno pre-immunized (Ad5/3Δ24, AdEpitopes and PeptiCRAd), the anti-viral response elicited in both AdEpitopes and in PeptiCRAd was statically lower compared to Ad5/3Δ24 (**Figure 5C**). This suggests that both approaches shifted the immune response from being mainly antiviral to being mainly tumor-antigenic specific, with PeptiCRAd showing the best performance in switching the immune response from antiviral to antigen-specific T cells (**Figure 5C**). Upon antigenic stimulation, we also detected the level of IL-10 in the cultures of splenocytes; a high release of IL-10 was observed in PeptiCRAd treated group upon TRP2 stimulus, suggesting that CD8+T cells were highly activated and cytolytic (32) (**Figure 5D**). Upon adeno stimulation, the highest production of IL-10 was instead reported in the Ad5/3Δ24 treated group, well in line with the previous observation that either AdEpitopes or PeptiCRAd shifted the immune response from mainly anti-viral to mainly anti-tumoral (**Figure 5D**). Strictly, the ratio IFN-γ/IL-10 was increased in AdEpitopes treated mice upon TRP2 stimulation compared to PeptiCRAd (**Figure 5E**), suggesting that PeptiCRAd induced more activated and cytolytic CD8+T cells compared to AdEpitopes as both IFN-γ and IL-10 showed upregulation in PepitCRAd (**Figure 5E**). The data demonstrated that we have first generated an OAd encoding TNAs that we used in turn to benchmark PeptiCRAd with main regard to its prophylactic efficacy. The results showed similar immune efficacy in eliciting a specific anti-tumor response, highlighting however that PeptiCRAd could induce more activated and cytolytic immunophenotypes in the CD8+ T cells population compared to AdEpitopes.

### PeptiCRAd Monotherapy Created a Systemic Anti-Tumor Immune Response Controlling the Tumor Growth of Distant Untreated Cancer Lesions in a Poorly Immunogenic Melanoma Model

**Figure 6:**
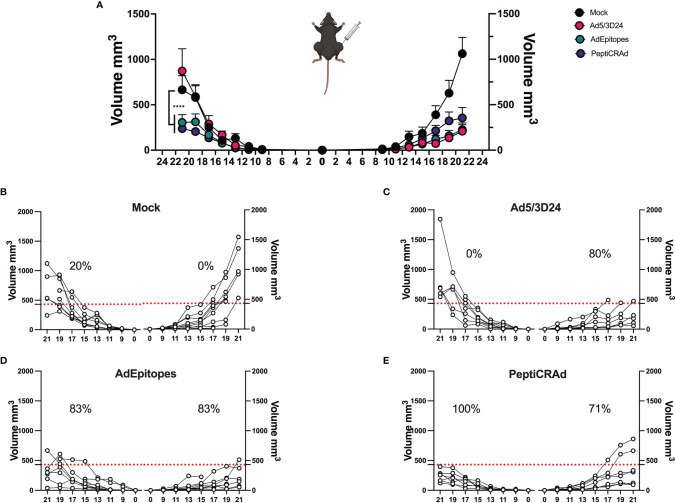
PeptiCRAd elicited local and systemic antitumor response in a poor immunogenic tumor melanoma model. **(A)** Immunocompetent C57Bl/6 mice were subcutaneously injected with the syngeneic tumor model B16.F1 in the left (0.5x10^4^ cells) and right flank (1x10^5^). Ad5/3Δ24, AdEpitopes and PeptiCRAd were intratumorally administrated four times, two days apart starting from day 9. The B16F1 tumor growth was followed until the end of the experiment and the tumor size is presented as the mean ± SEM and statistically difference was assessed with two-way ANOVA; (ns P > 0.05, *P ≤ 0.05, **P ≤ 0.01, ***P ≤ 0.001, ****P ≤ 0.0001). **(B–E)** Single tumor growth for single mouse for Mock **(B)**, Ad5/3Δ24 **(C)**, AdEpitopes **(D)** and PeptiCRAd **(E)** is shown. A threshold of 431 mm^3^ was set to define the percentage of mice responding to the different therapies (red dotted line) for right and left tumors. The percentage of responders in each treatment group is shown on the top of the dotted line. The threshold was defined as the median of the tumor size at the last day of the experiment in Ad5/3Δ24 treated group. (Mock n=9, Ad5/3 Δ24n=9, AdEpitopes n= 9, PeptiCRAd n=8).

**Figure 7:**
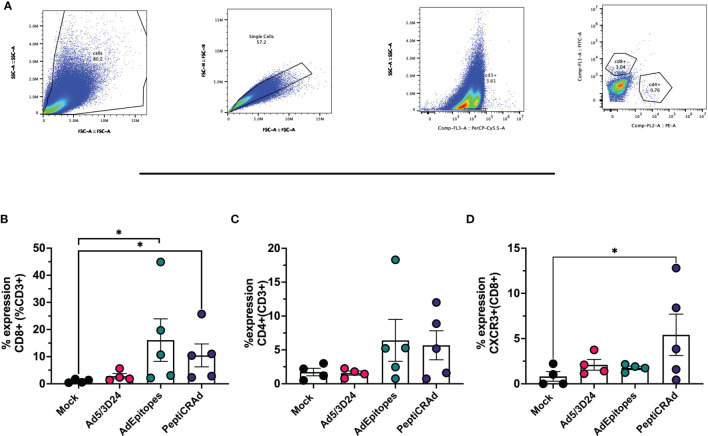
PeptiCRAd induced immune infiltration in distant not treated cancer lesions. **(A)** A representative gating strategy for the flow cytometry analysis of the untreated tumors is showed. **(B–D)** The immunological T cell profile was investigated in untreated lesions by flow cytometry. The frequency of CD8+ **(B)**, of CD4+ **(C)** and CXCR3+ (CD8+) **(D)** T cells is shows. All the data are plotted as dot plot for each tumor and for each treatment group as mean± SEM. The significance was assessed by One way ANOVA and Tukey´s correction (ns P > 0.05, *P ≤ 0.05, **P ≤ 0.01, ***P ≤ 0.001, P ≤ 0.0001).

We have demonstrated that PeptiCRAd technology elicited a specific anti-tumor immune response at a similar magnitude to cloned viruses regarding its prophylactic activity. Next, we wanted to assess whether PeptiCRAd could work as a therapeutic approach also in the context of TNAs. To this end, immunocompetent C57Bl/6 mice were subcutaneously injected with the syngeneic B16F1 melanoma cells in the right and left flanks. When the tumors were established, AdEpitopes or PeptiCRAd counterpart were injected intratumorally only in the right tumors. Mock and Ad5/3Δ24 were used as controls; the viral dose used was 1x10^9^ VP/tumor. At the end of the experiment, AdEpitopes and PeptiCRAd showed tumor growth control in the treated lesions with the same efficacy of Ad5/3Δ24 (**Figure 6A**) as depicted also in the single tumor growth per each mouse per each treatment group (**Figures 6B–E**), indicating local anti-tumor activity due mainly to Ad5/3Δ24. However, both AdEpitopes and PeptiCRAd counterparts, but not Ad5/3Δ24 slowed down the tumor growth of the not-injected lesion (left side) (**Figure 6A**), highlighting that both approaches elicited systemic anti-tumor specific response. These observations prompted us to further investigate the immune components within the untreated lesions (left side). First, we observed a statically relevant increased infiltration of CD8+ T cells in both AdEpitopes and PeptiCRAd treated mice (**Figures 7A, B**); moreover, as both approaches also engaged MHC-II restricted epitopes, accordingly a tendency in increased CD4+T (**Figure 7C**) cells was also observed. The migratory marker CXCR3 was in general downregulated in the CD8+T cells population, whereas PeptiCRAd statically increased the CXCR3+CD8+T cells within the TME of the untreated lesions (**Figure 7D**).

Altogether, the data confirmed that PeptiCRAd monotherapy effectively exerts anti-tumoral activity in the context of TNAs through modulation of the immune response, in particular modulating the adaptive immune response (CD4+ and CD8+ T cell population). PeptiCRAd acted as efficiently as a traditional OAd encoding TNAs, acting as de facto cancer vaccines.

In conclusion, we have exploited a “plug-and-play” technology named PeptiCRAd, based on decorating OVs (OAd) with tumor peptides to elicit specific anti-tumor T cell response and we have carefully analyzed this technology in comparison to conventional oncolytic cancer vaccines. Indeed, we combined the viral immunogenicity with tumor peptides to guide the immune response specifically to the eradication of cancer. Compared to conventional OAd encoding tumor antigens, PeptiCRAd showed comparable efficacy in both prophylactic and therapeutic profiles. Moreover, PeptiCRAd showed anti-tumor efficacy in the context of TNAs, generating a systemic anti-tumor response to the same extent as the counterpart conventional cancer OV. However, PeptiCRAd retains the advantage of being rapidly adapted by coating the adenovirus with a new set of tumor antigens, a crucial key in personalized cancer vaccines clinical setting and therefore PeptiCRAd can be considered a valid alternative to OAd encoding TAs.

## Discussion

In this work, we have investigated PeptiCRAd, a “plug-and-play” technology based on OAd coated with tumor peptides to assess its prophylactic and therapeutic efficacy in comparison to conventional OAd encoding TAs, that currently represent one of the most exploited platforms in the field for cancer vaccines due to its immunogenicity and tumor cell lysis capability (33). PeptiCRAd consists of an OAd coated through electrostatic interactions with positively charged MHC-I restricted tumor peptides (poly-lysine tail-peptides) (26). The reaction requires only 15 minutes and several studies have shown its anti-tumor efficacy and immunological modulation in different contexts and tumor models such as murine triple-negative breast cancer (34), as a cancer therapeutic platform for immunopeptidomic pipelines (7, 35), as a tool to explore viral mimicry to tumor antigens for cancer immunotherapy (36), as a platform to exploit pre-existing immunity to pathogens for boosting anti-tumoral CD8 T cell response (37) or to decorate with tumor peptides OAd encoding immunostimulatory molecules (38). However, we have never compared our cancer vaccine platform with traditional approaches in the field such as OAd genetically modified to encode TAs. Therefore, here we have aimed to confront the immunological modulation and anti-tumor profile of PeptiCRAd with OAd encoding TAs. To mimic clinically relevant context, we have generated and characterized *in-house* OAd encoding TAs. As the production of novel OAd encoding TAs requires validation and characterization procedure, for each cloned OAd we have always investigated the oncolytic activity and the transgene expression at both RNA and protein level whenever it was technically possible. Indeed, the manufacture of cancer therapeutic vaccines is still facing the limitation of identifying a delivery system that is cost-effective and timely convenient (39, 40). In contrast, PeptiCRAd is a cloning-free technology that relies on coating OAd with tumor peptides, making it easily adaptable to personalized cancer medicine. Additionally, upon advancements in manufacturing and manipulation, the peptides are relatively less expensive and due to their synthetic nature, batch-to-batch variation is avoided (41). However, the immunogenicity of peptides is limited, and several strategies have been explored to enhance their efficacy (42, 43). PeptiCRAd offers a solution for both time-demanding vaccine platform development and the weak immunogenicity of peptide-based therapy thanks to fast electrostatic interaction and the adenoviral immunostimulatory properties. Through this work, we have adopted two main strategies for the comparative analysis: *in vitro* stimulation of murine dendritic cell line and *in vivo* murine vaccination studies. The proof of concept has exploited the tumor antigen OVA because several tools and specific antibodies are available for the downstream detection of this antigen; the *in vitro* study highlighted that both AdOVA and PeptiCRAd have elicited the presentation of TAs in an immunogenic fashion as shown by the contemporaneity expression of H2Kb-bound to SIINFEKL and upregulation of CD86 molecule; in details, PeptiCRAd induced immunogenic modulation already 24h post-infection, whereas AdOVA required 48h. The different kinetic is explained by the nature of the two platforms. The cloned virus´ antigen expression relies on the translation of the construct, in contrast, PeptiCRAd directly delivers the peptides to DCs, priming the APCs faster and avoiding issues related to the genetic expression of TAs. Indeed, a well-known bottleneck in the application of OVs encoding TAs as cancer therapeutic vaccines is that transgene expression depends on viral genome replication. Indeed, this latter requires robust viral infection and ultimately the vaccination depends on the unpredictable and highly variable intrinsic sensitivity of each tumor to OVs (25). Once we showed that the priming of DCs in *in vitro* experimental settings was comparable at least under a quality point of view between AdOVA and PeptiCRAd, we needed to further explore the licensing and generation of antigen-specific T cells upon DC activation. To this end, the adaptive immune response was assessed in *in vivo* by preimmunization of mice. The functional characterization of T cell response confirmed that vaccination with PeptiCRAd could induce antigen-specific T-cells response to the same extent as cloned viruses and, most importantly, our proof of concept has shown the same results also with the clinically relevant tumor antigen TRP2_180-188_. However, to compare the anti-tumoral efficacy with OAd encoding TAs, our technology was used as a therapeutic approach for the treatment of tumor-bearing mice. The tumor growth was slowed down in PeptiCRAd as well as in AdTRP2, confirming that our technology has activated DCs that in turn have primed and generated specific T-cells response; these latter have then exercised anti-tumor activity. The first part of our work has confirmed that our technology worked similarly to a conventional adeno-based cancer vaccine. Next, we have moved to a more relevant clinical scenario, involving the use of TNAs for cancer therapeutic approaches. Indeed, in cancer immunotherapy TNAs have gained momentum, becoming the preferential target of several therapeutic strategies. TNAs are selectively expressed in cancer cells, minimizing immune tolerance as well as autoimmune reactions (44); additionally, TNAs are more likely to engage CD8+ T-cell response and in this sense, the cancer immunotherapy could exploit personalized treatments, taking into account cancer patients´ mutanome for a rational design of cancer therapeutic vaccine (45–47). To answer this need, we have applied our technology for the targeting of five TNAs found in the murine cell line B16F1, as previously described (27). Herein, we have generated an OAd encoding the five TNAs and then performed an *in vivo* characterization in mice to test both the prophylactic and the therapeutic efficacy. The prophylactic results have confirmed the efficacy of both approaches in generating specific T- cell response and, interestingly, the detection of IL-10 in mice pre-immunized with PeptiCRAd indicated highly activated and cytolytic CD8+ T-cells (32). This observation was well in line with the anti-tumor effect observed in mice bearing the tumor melanoma B16F1. Interestingly, both AdEpitopes and PeptiCRAd controlled the tumor growth of both the injected and not-injected lesions. This effect is known as “abscopal effect” and it indicated the generation of a systemic specific anti-tumor response able to eradicate distant lesions (48). The data were then confirmed by subsequent immunological analysis, showing an increased infiltration of effector CD8+ T cells within the TME.

In summary, PeptiCRAd could serve as a platform for cancer oncolytic vaccines, as we showed that the immunological profile and the anti-tumor activity are comparable to conventional cloned-based adenoviral platform vaccines. In addition, PeptiCRAd offers an easy and time-effective solution for the generation of therapeutic vaccines. Secondly, the antigen is delivered as a peptide and it is readily available to APCs, meaning that the issues related to genetic expression are avoided. Third, PeptiCRAd can be easily customized depending on the patient´s specific mutations and tumor development. Finally, as OAd is decorated with peptides, the viral genome can be modified to express transgenes such as immunostimulatory molecules (i.e.GM-CSF). Ultimately, we could combine in a single platform, viral immunogenicity with a specific antitumor response guided by the peptides and an enhanced immune activation due to the expression of immunostimulatory molecules encoded in the viral genome.

## Data Availability Statement

The original contributions presented in the study are included in the article/**Supplementary Material**. Further inquiries can be directed to the corresponding author.

## Ethics Statement

All animal experiments were reviewed and approved by the Experimental Animal Committee of the University of Helsinki and the Provincial Government of Southern Finland (license number ESAVI/11895/2019).

## Author Contributions

SF: conception and design, acquisition of data, analysis and interpretation of data, drafting of manuscript. SR: conception and design, acquisition of data, analysis and interpretation of data, drafting of manuscript. BM: conception and design, analysis and interpretation of data, drafting of manuscript. AL: acquisition of data, analysis and interpretation of data. GV: acquisition of data, analysis and interpretation of data. VF: revising of manuscript. CG.: analysis and interpretation of data. MF: interpretation of data, revising of manuscript. SP: interpretation of data, revising of manuscript. EY: analysis and interpretation of data, revising of manuscript. MiF: analysis and interpretation of data, revising of manuscript. SP: interpretation of data, revising manuscript. GA: interpretation of data, revising manuscript. JC: interpretation of data, revising of manuscript. FH: interpretation of data, revising of manuscript. MF: conception and design, interpretation of data, revising of manuscript. MG: conception and design, interpretation of data, revising of manuscript. VC: conception and design, acquisition of data, analysis and interpretation of data, drafting of manuscript. All authors contributed to the article and approved the submitted version.

## Funding

This work has been supported by the European Research Council under the European Union’s Horizon 2020 Framework program (H2020)/ERC-CoG-2015 Grant Agreement No. 681219, the Helsinki Institute of Life Science (HiLIFE), the Jane and Aatos Erkko Foundation (decision 19072019), the Cancer Society of Finland (Syöpäjärjestöt).

## Conflict of Interest

VC is a co-founder and shareholder at VALO Therapeutics. SP is an employee and a shareholder at VALO Therapeutics.

The remaining authors declare that the research was conducted in the absence of any commercial or financial relationships that could be construed as a potential conflict of interest.

## Publisher’s Note

All claims expressed in this article are solely those of the authors and do not necessarily represent those of their affiliated organizations, or those of the publisher, the editors and the reviewers. Any product that may be evaluated in this article, or claim that may be made by its manufacturer, is not guaranteed or endorsed by the publisher.
