# Supplementary material for: Peptides-Coated Oncolytic Vaccines for Cancer Personalized Medicine
Source: Front Immunol. 2022 Apr 14;13:826164. doi: 10.3389/fimmu.2022.826164 (PMC9047942; doi:10.3389/fimmu.2022.826164)
Supplement: Supplementary file 1 [file DataSheet_1.docx]

**Supplementary Figures and Supplementary Figure Legends**


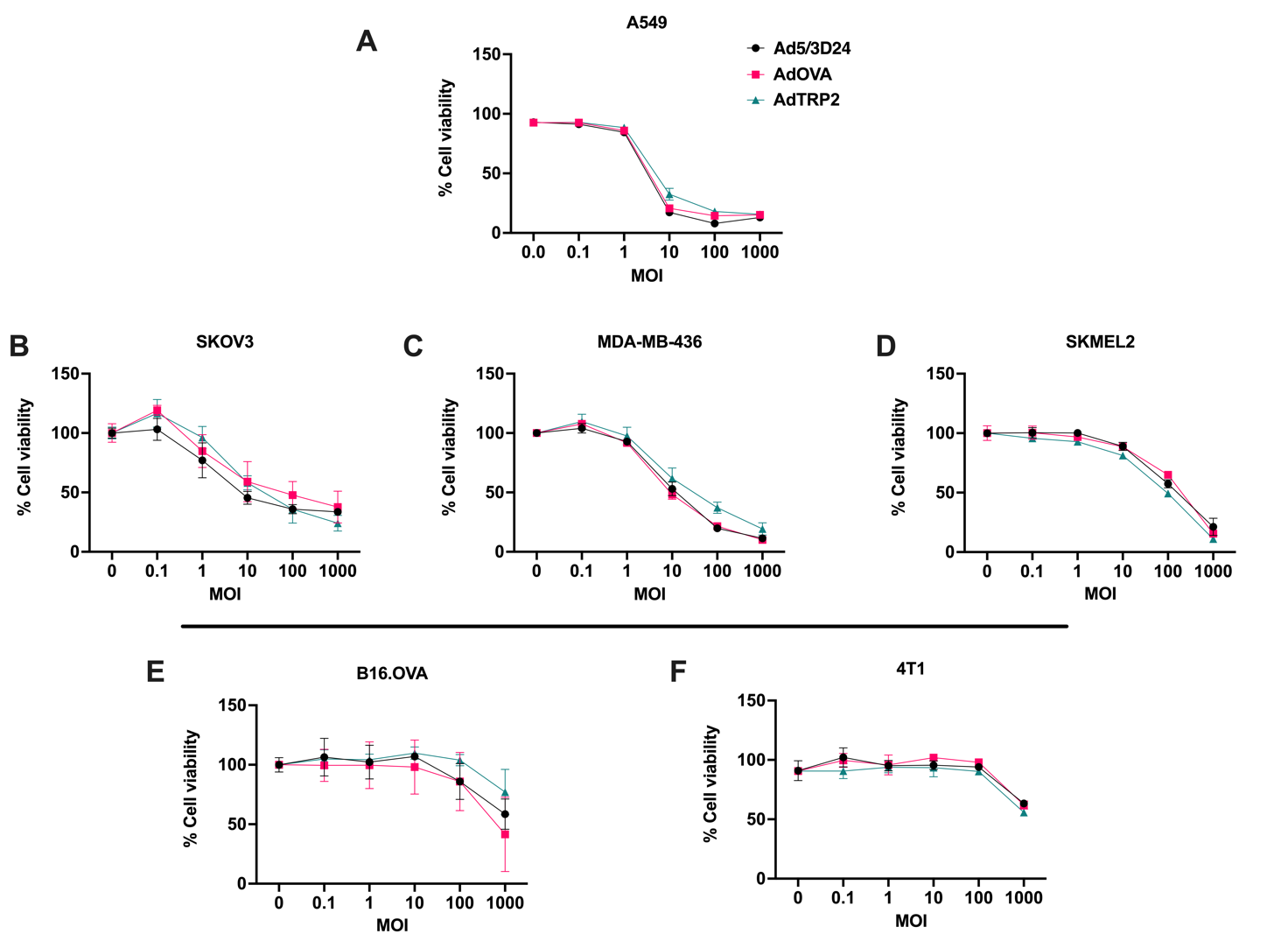


**Supplementary Figure 1** Cell viability in A549 (**A**), SKOV3 (**B**), MDA-MB-436 (**C**) and SKMEL-2 (**D**) and in two murine cell lines B16.OVA (**E**) and 4T1 (**F**) was evaluated using (3-(4,5-dimethylthiazol-2-yl)-5-(3-carboxymethoxyphenyl)-2-(4-sulfophenyl)-2H-tetrazolium) (MTS) assay. Infection with Ad5/3Δ24 (black), AdOVA (pink) or AdTRP2 (green) was done at different MOI as indicated in the figure and the viability was evaluated at 3days post infection. The data are shown as mean ± SEM.

**
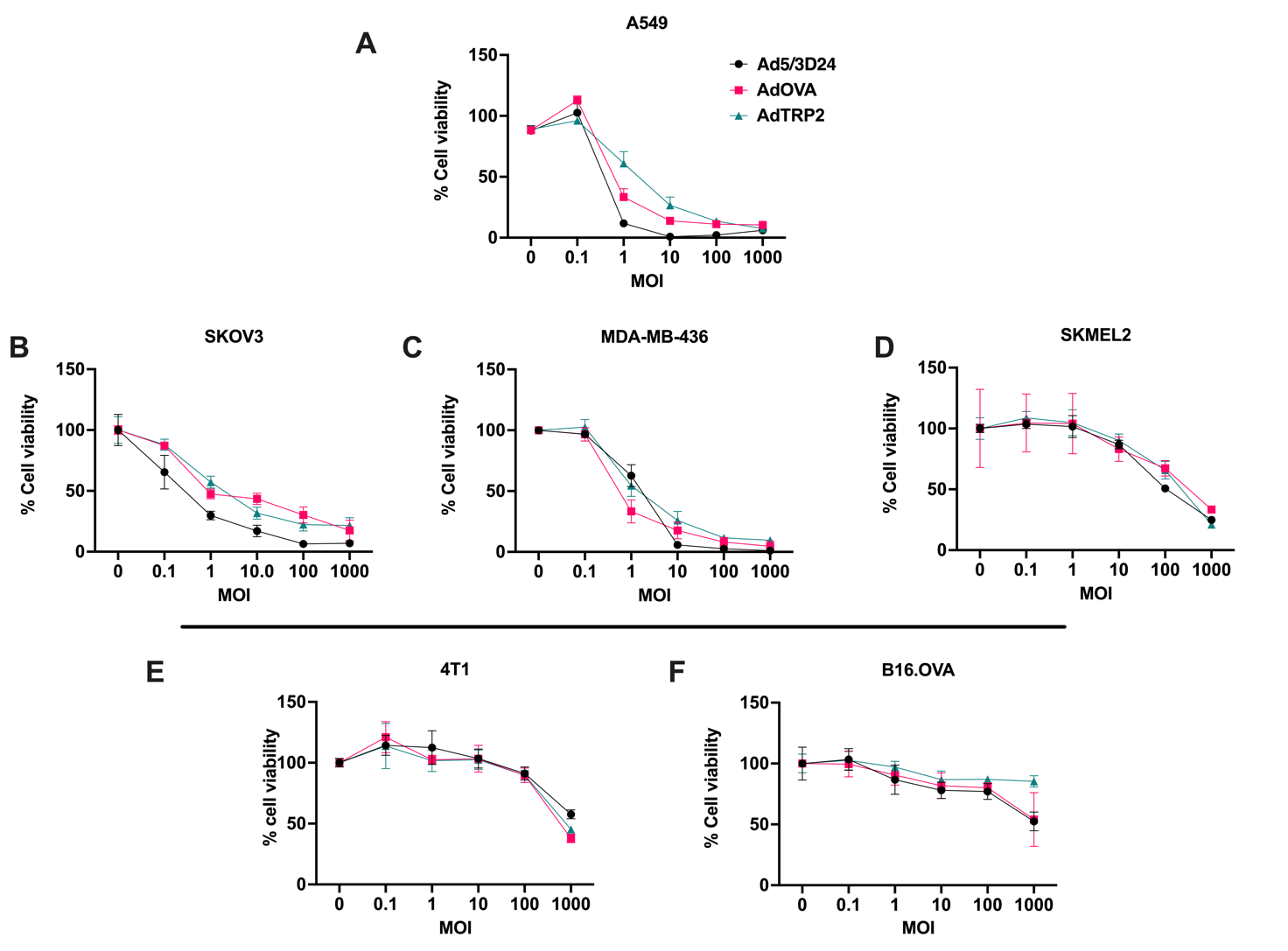
**

**Supplementary Figure 2** Cell viability in A549 (**A**), SKOV3 (**B**), MDA-MB-436 (**C**) and SKMEL-2 (**D**) and in two murine cell lines B16.OVA (**E**) and 4T1 (**F**) was evaluated using (3-(4,5-dimethylthiazol-2-yl)-5-(3-carboxymethoxyphenyl)-2-(4-sulfophenyl)-2H-tetrazolium) (MTS) assay. Infection with Ad5/3Δ24 (black), AdOVA (pink) or AdTRP2 (green) was done at different MOI as indicated in the figure and the viability was evaluated at 5 days post infection. The data are shown as mean ± SEM.

**
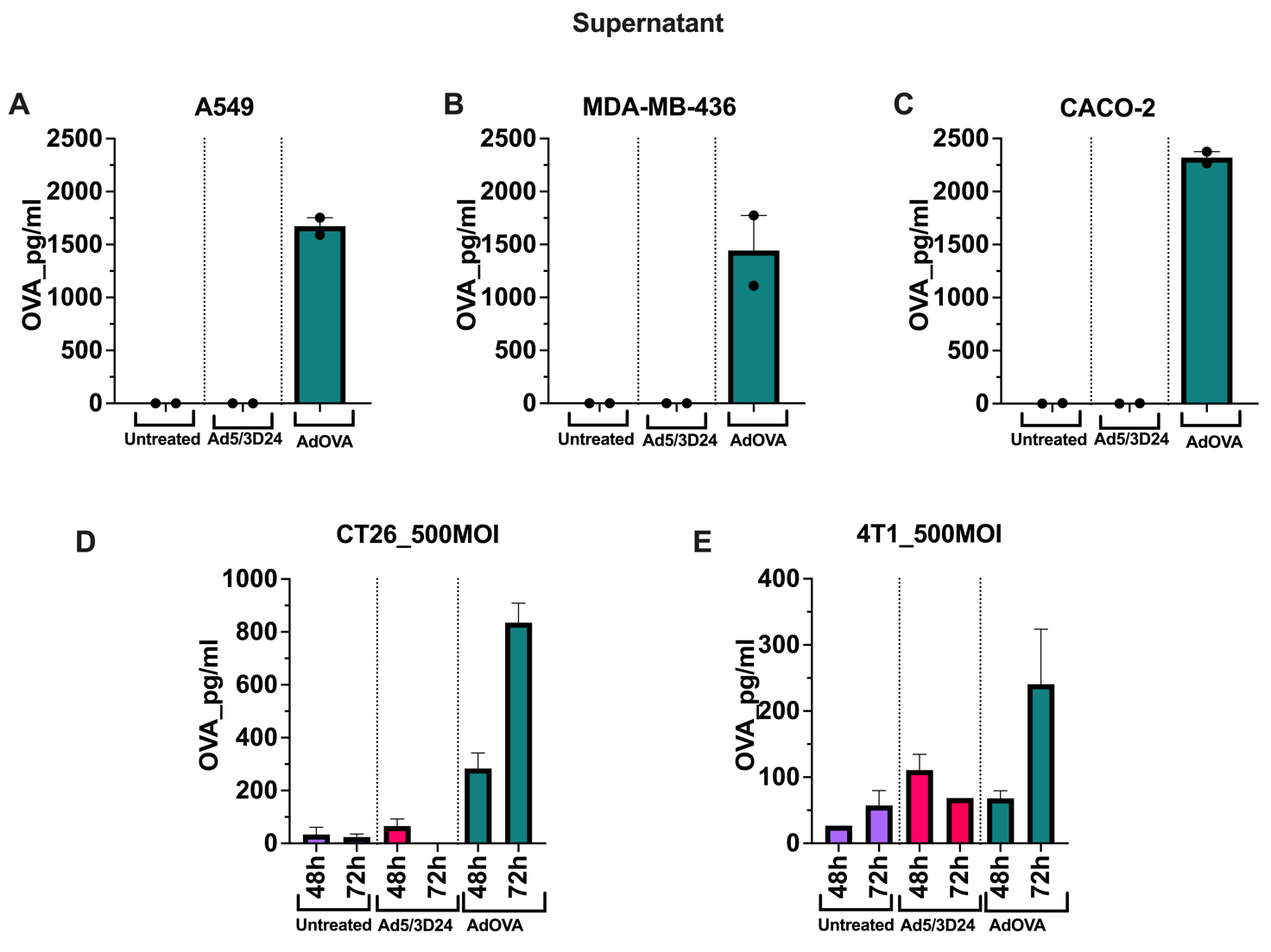
**

**Supplementary Figure 3** The level of OVA protein was detected in the supernatant of three human cell line A549 (**A**), MDA-MB-436 (**B**) and CACO-2 (**C**) and in the supernatant of two murine cell line CT26 (**D**) and 4T1 (**E**) at 48h and 72h post infection. The data are depicted as bar plot mean± SEM.

**
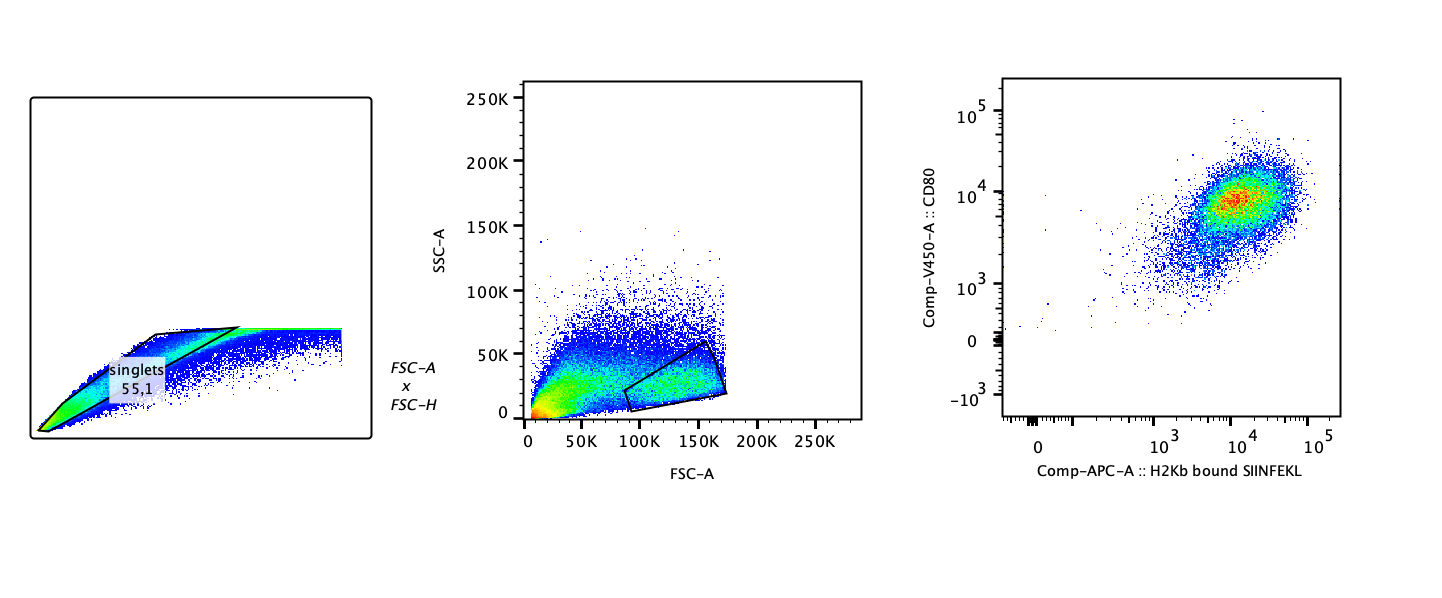
**

**Su**

**Supplementary Figure 4** A representative gating strategy for the flow cytometry analysis of the mouse dendritic cell line JAWS II pulsed with Ad5/3Δ24, peptide alone (polyKSIINFEKL), AdOVA, PeptiCRAd-SIINFEKL or left unpulsed (cells only) is reported.

**
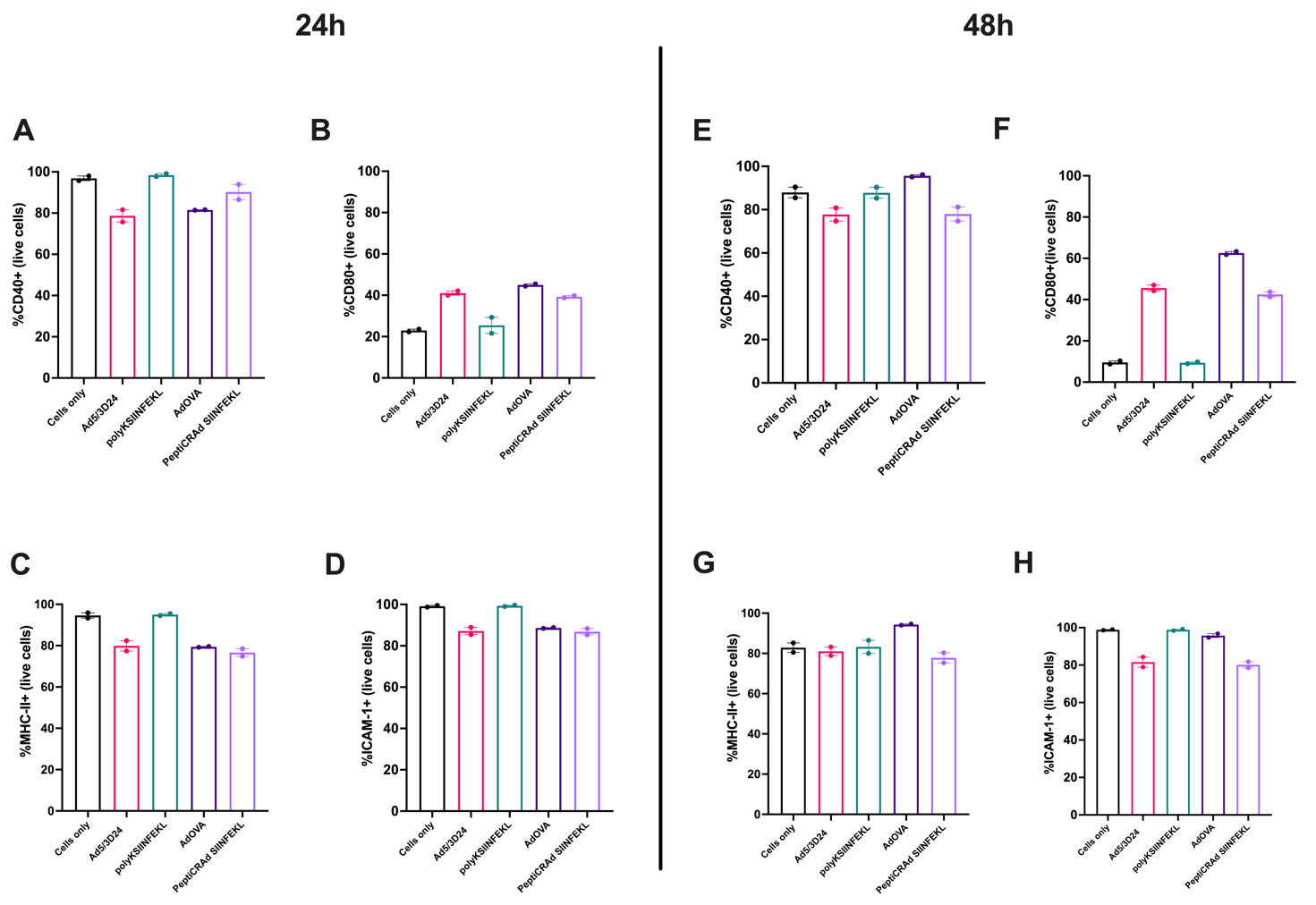
**

**Supplementary Figure 5** Mouse dendritic cell line JAWS-II was pulsed with Ad5/3Δ24, peptide alone (polyKSIINFEKL), AdOVA, PeptiCRAd-SIINFEKL or left unpulsed (cells only). The surface markers CD40 (**A**), CD80 (**B**), MHC-II (**C**) and ICAM-I (**D**) were determined by flowcytometry at 24h (left panel). The same surface markers were evaluated also at 48 h, CD40 (**E**), CD80 (**F**), MHC-II (**G**) and ICAM-I (**H**) (right panel). The data are shown bar plot mean± SEM.


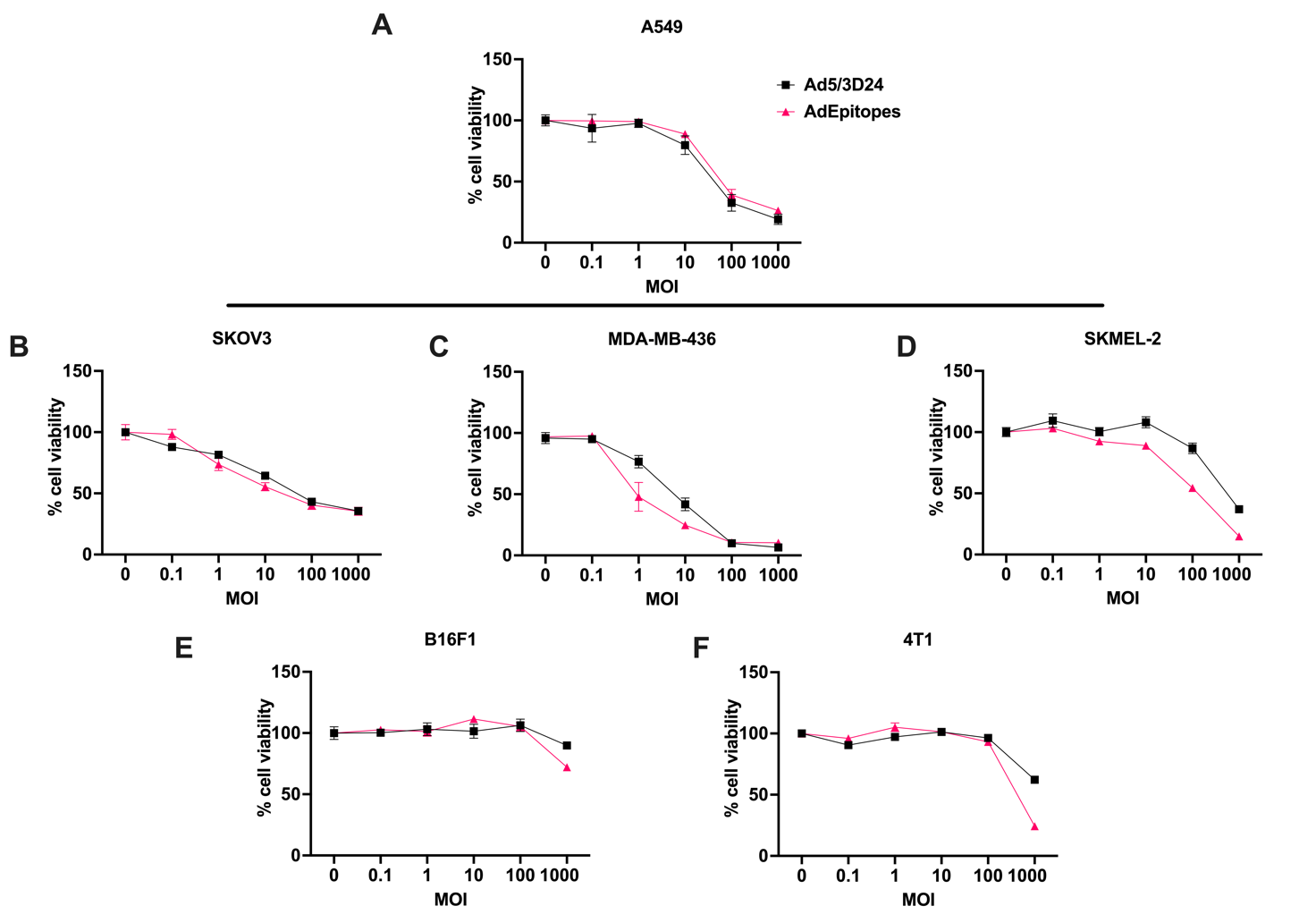


**Supplementary Figure 6** Cell viability in A549 (**A**), SKOV3 (**B**), MDA-MB-436 (**C**) and SKMEL-2 (**D**) and in two murine cell lines B16F1 (**E**) and 4T1 (**F**) was evaluated using (3-(4,5-dimethylthiazol-2-yl)-5-(3-carboxymethoxyphenyl)-2-(4-sulfophenyl)-2H-tetrazolium) (MTS) assay. Infection with Ad5/3Δ24 (black), AdOVA (pink) or AdTRP2 (green) was done at different MOI as indicated in the figure and the viability was evaluated at 3 days post infection. The data are shown as mean ± SEM.


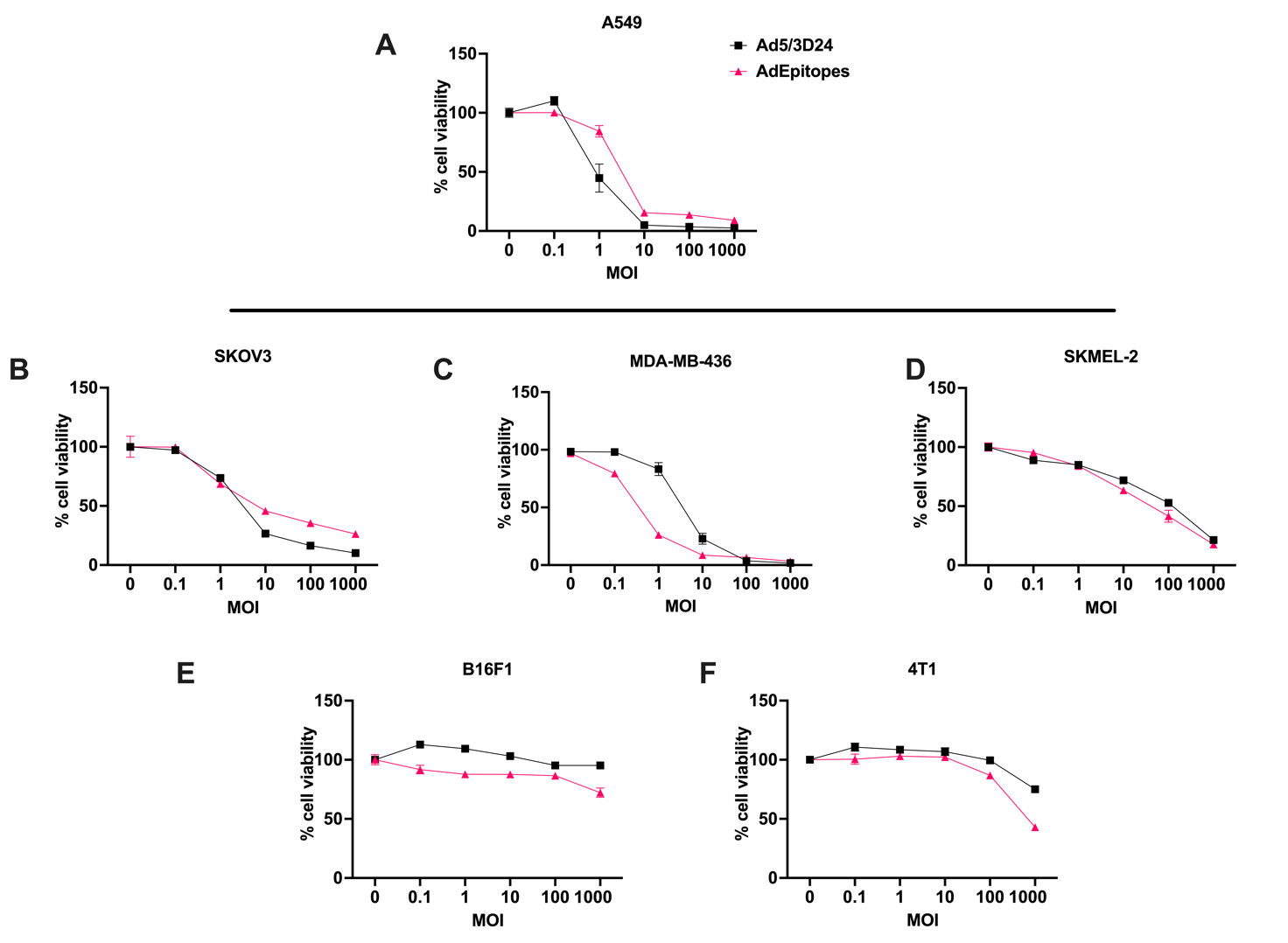


**Supplementary Figure 7** Cell viability in A549 (**A**), SKOV3 (**B**), MDA-MB-436 (**C**) and SKMEL-2 (**D**) and in two murine cell lines B16F1 (**E**) and 4T1 (**F**) was evaluated using (3-(4,5-dimethylthiazol-2-yl)-5-(3-carboxymethoxyphenyl)-2-(4-sulfophenyl)-2H-tetrazolium) (MTS) assay. Infection with Ad5/3Δ24 (black), AdOVA (pink) or AdTRP2 (green) was done at different MOI as indicated in the figure and the viability was evaluated at 5 days post infection. The data are shown as mean ± SEM.
